# Is DNA Barcoding Actually Cheaper and Faster than Traditional Morphological Methods: Results from a Survey of Freshwater Bioassessment Efforts in the United States?

**DOI:** 10.1371/journal.pone.0095525

**Published:** 2014-04-22

**Authors:** Eric D. Stein, Maria C. Martinez, Sara Stiles, Peter E. Miller, Evgeny V. Zakharov

**Affiliations:** 1 Southern California Coastal Water Research Project, Costa Mesa, California, United States of America; 2 Biodiversity Institute of Ontario, University of Guelph, Guelph, Ontario, Canada; University of Milan-Bicocca, Italy

## Abstract

Taxonomic identification accounts for a substantial portion of cost associated with bioassessment programs across the United States. New analytical approaches, such as DNA barcoding have been promoted as a way to reduce monitoring costs and improve efficiency, yet this assumption has not been thoroughly evaluated. We address this question by comparing costs for traditional morphology-based bioassessment, the standard Sanger sequencing-based DNA barcoding approach, and emerging next-generation (NGS) molecular methods. Market demand for molecular approaches is also assessed through a survey of the level of freshwater bioassessment effort in the United States across multiple habitat types (lakes, streams, wetlands) and indicators (benthic invertebrates, fish, algae). All state and regional level programs administered by public agencies and reported via agency web sites were included in the survey. Costs were based on surveys of labs and programs willing to provide such information. More than 19,500 sites are sampled annually across the United States, with the majority of effort occurring in streams. Benthic invertebrates are the most commonly used indicator, but algae and fish comprise between 35% and 21% of total sampling effort, respectively. We estimate that between $104 and $193 million is spent annually on routine freshwater bioassessment in the United States. Approximately 30% of the bioassessment costs are comprised of the cost to conduct traditional morphology-based taxonomy. Current barcoding costs using Sanger sequencing are between 1.7 and 3.4 times as expensive as traditional taxonomic approaches, excluding the cost of field sampling (which is common to both approaches). However, the cost of NGS methods are comparable (or slightly less expensive) than traditional methods depending on the indicator. The promise of barcoding as a cheaper alternative to current practices is not yet realized, although molecular methods may provide other benefits, such as a faster sample processing and increased taxonomic resolution.

## Introduction

Bioassessment has become a cornerstone of environmental monitoring over the past 30 years. Since Karr [1] first introduced the concept for fish in streams, bioassessment has expanded worldwide to include multiple aquatic habitats (streams, estuaries, lakes, wetlands) and multiple indicator groups (invertebrates, algae, plants, and amphibians; [2]. Most states in the U.S. use bioassessment to support State water quality assessment programs, to comply with Federal mandates and (to a lesser extent) as a regulatory tool to assess compliance with biological objectives or criteria [3].

Bioassessment is the use of biological community composition as an indicator of condition or stress; this is attractive because resident organisms integrate the influences of environmental conditions over time and space and therefore can be more indicative of overall environmental health than measuring individual stressors or ecosystem attributes [4]. More recently, regulatory programs are developing biologically based standards, objectives or criteria that use biological indices as a basis for permit compliance, restoration success, or stressor remediation. The increased application of bioassessment has translated to an increased need for local capacity for taxonomic identification and interpretation. This need is complicated by the fact that sufficient numbers of trained taxonomists are rarely available and experience, quality, and level of taxonomic resolution varies considerably across states and programs [5].

Among the concerns associated with expansion of bioassessment programs are the time and cost associated with obtaining monitoring results. It may take six months or longer following field collection to get taxonomic data that can be used to generate the biological indices used for management or regulatory decision making. This may be acceptable for routine ambient monitoring programs; however, for assessing regulatory compliance this time lapse means that results indicate conditions that existed 6–9 months earlier. Expedient or tactical regulatory management responses are difficult given this time lag. Furthermore, the analytical cost for conducting bioassessment at a single site can be in excess of $500 (US), making exploratory assessments difficult to justify.

Molecular methods, such as DNA barcoding, are increasingly proposed as tools to enhance bioassessment capacity by reducing the time and cost necessary for taxonomic identification and therefore addressing some of the challenges associated with expanding programs [6]–[8]. Barcoding involves identifying taxa based on a short DNA sequence from a standardized genetic locus, such as the metazoan-targeted mitochondrial gene cytochrome c oxidase I (COI). Using standard molecular methods, DNA is extracted from a specimen tissue, amplified using universal primers, and, in case of animal species, sequenced for the approximately 650-bp “barcode” region of COI [9]. DNA from unknown specimens collected in benthic samples can be identified by comparing their barcode sequences to a nucleotide sequence reference library, such as the Barcode of Life Data Systems (BOLD; www.boldsystems.org) [10] or GenBank (www.ncbi.nlm.nih.gov/genbank).

Predictions of increased speed and reduced cost of DNA barcoding relative to traditional bioassessment approaches are largely speculative. Rigorous evaluation of any increases in efficiency requires consideration of both relative cost of molecular vs. morphological approaches and overall market demand for a new approach. Analysis of relative cost should consider all steps of the bioassessment and barcoding processes including sorting, picking out debris, specimen identification, vouchering, DNA extraction, PCR amplification and sequencing. For example, if a specific step of sample processing would be required regardless of whether morphological or molecular methods were used, it should not be part of the evaluation of cost or time tradeoffs. Consideration of time and costs should be made in the context of “real-world” applications and include programs with different biological endpoints (e.g. fish, invertebrates, algae) that may have different constraints in terms of cost and processing time.

New methods require investment in new infrastructure. If overall demand is high, even incremental reductions in cost or processing time can translate to large increases in efficiency and therefore motivate the investment necessary to make the transition. Assessing the relative benefits of DNA barcoding should include a broad scale understanding of the level of effort currently devoted nationally to bioassessment for various indicators and waterbody types. Twelve years ago Carter and Resh [11] estimated that between 13,000 and 15,000 benthic invertebrate samples were collected from streams and processed each year by state programs. Comparable information is not readily available for other taxa (e.g. fish, algae) or for other habitats (e.g. wetlands, lakes).

We attempt to determine the potential for improved efficiency associated with a transition to molecular based bioassessment for the United States by 1) analyzing the time and cost associated with each step of the freshwater bioassessment process so that relative costs of morphological vs. molecular approaches can be compared based on their analogous steps; 2) comparing relative costs or time differences between traditional morphology-based approaches and application of DNA barcoding using Sanger sequencing-based methods; and 3) evaluating market demand by enumerating ongoing freshwater bioassessment efforts in all major aquatic environments in the U.S.

## Methods

### Relative cost of traditional vs. molecular based taxonomy

Two criteria were used to compare the relative “efficiency” of DNA barcoding to morphology-based taxonomy. First, we estimated the time required to process samples to an endpoint where they can be used for calculating bioassessment metrics. For the morphologic approach, this is a list of specimens identified to the standard taxonomic level (typically genus or species). Bioassessment sampling is comprised mainly of Arthropods, but may include other taxa such as Molluscs or Nematodes. For the barcoding approach, this is a list of sequences and associated organisms in a sequence reference library that meet quality requirements of a Barcode Identification Number (BIN) system [10] or can be used in species delimitation analysis (typically shorter sequences). Second, we estimated the cost necessary to generate the endpoint for each method (mentioned above), exclusive of the actual field sampling.

Relative per site costs in terms of time/labor and dollars were estimated based on traditional morphology and molecular analysis. Comparisons were made based on cost information from 3 and 6 labs in California (depending on the indicator and the habitat) and validated using a side by side comparison of samples collected as part of a watershed assessment in southern California, USA. Twelve sites were sampled for benthic macroinvertebrates in the San Gabriel watershed representing a range of conditions from pristine mountain streams to urban flood control channels. Field sites were either within the Angeles National Forest (U.S. Forest Service) or within the flood control right of way of Los Angeles County Flood Control District and its participating cities. Permits and access permissions were granted by the relevant agency. All field personnel possessed valid Scientific Collecting Permits from the California Department of Fish and Wildlife which authorizes collection for scientific, educational, and non-commercial propagation purposes.

Cost validation was based on standard protocols used in California, and elsewhere. Benthic macroinvertebrates (BMIs) were sampled using the multihabitat method described by Ode [12]. Each 150-m segment was divided into 11 equidistant transects, and a 500 µm mesh D-frame net was used to collect BMIs from a prescribed location along each transect (i.e., 25, 50, or 75% of the way across the stream), for a total of 0.9 m^2^ of streambed sampled. The 11 subsamples were composited into one container and specimens were preserved immediately using 95% ethanol. Samples were drained and replenished with fresh ethanol within 24 hours of collection to maintain a minimum 90% ethanol concentration to prevent DNA degradation. A minimum of 600 BMIs were sorted and identified in the laboratory based on standard protocols and following the taxonomic standards of the Southwestern Association of Freshwater Invertebrate Taxonomists (i.e., level 2 in Richards and Rogers) [13].

For DNA barcoding, following traditional taxonomic identification, samples were treated in two ways. For about half of the specimens, (comprised of non-chironomids (Arthropoda: Insecta: Diptera)) whole vouchers were sent to the Canadian Center for DNA Barcoding (CCDB) where they underwent complete processing including tissue subsampling into 96-well plates (and, where required, voucher recovery), voucher imaging, and automated DNA extraction followed by routine barcoding analysis [14] and http://www.ccdb.ca/resources.php). For the remaining half of the specimens, comprised of all the chironomids, subsampling and DNA extraction (following CCDB protocols) was completed prior to shipment and the resulting DNA extracts were shipped to CCDB for further barcode analysis including one or two rounds of PCR, and bidirectional Sanger sequencing. Sequences and detailed information about all specimens were uploaded to the Barcode of Life Data Systems [10] and are accessible via the BOLD project code SGABR.

Field sampling costs are the same regardless of approach; however, specimen analysis costs will vary between morphological and molecular methods. For benthic invertebrates, we document costs based on labor for specimen sorting and on standard laboratory taxonomy rates. In California, approximately five laboratories do the majority of taxonomic identification; rates for these laboratories were comparable and our costs are representative. For indicators not included as part of the San Gabriel watershed study (e.g. algae and fish), taxonomy costs were provided by the contract laboratories typically used for those identifications.

Cost and time estimates for the molecular analysis were estimated based on two different approaches, single specimen Sanger sequencing of individual organisms and next-generation sequencing of composite DNA extracts from bulk samples of organisms using the IonTorrent PGM platform. The former is more commonly used, is likely more accurate and provides the ability to directly connect individual organisms and their DNA sequences to vouchered reference sequences. The latter is a platform for newer emerging sequencing technology, is less expensive, but arguably is less accurate. The Sanger sequencing approach was evaluated based on a three-step process. For the first step, we documented labor costs for sorting specimens, clipping tissues and placing them in 96-well plates. Costs for the second and third steps were provided by the CCDB, which is representative of costs that would be charged by commercial labs. The second step included labor and supplies for the DNA extraction and amplification phases. The third step included costs for sequencing, error checking, and second round sequencing (if necessary). The NGS approach does not involve placing tissues into individual wells, rather all the DNA from a bulk sample is extracted and amplified using a standard set (or sets) of primers. Therefore costs were estimated for the extraction and amplification phase and then for the sequencing and error checking phase.

### Assessment of market demand based on national level of effort

We compiled information on state/regional ongoing bioassessment programs in the United States as of the 2010–2012 time frames using a combination of internet searches and personal contact with program managers (Table 1 and 2). Information was verified through published reports or confirmation via personal interviews with agency representatives. We focused on regional and statewide ambient assessment programs that are facilitated or conducted through ongoing agency programs. We included programs focusing on all freshwater aquatic resource types that use fish, benthic invertebrates, or algae, as these are the most commonly used taxa for bioassessment. This approach expands on the survey conducted by Carter and Resh [11] who focused solely on U.S. state agencies that collect and process benthic macroinvertebrate samples from streams. City, county, or other local programs were not included given the sheer number that potentially occur nationally. Compliance monitoring or citizen science programs were not included because of the difficulty in obtaining reliable information about these programs and their somewhat transient nature. Our estimates represent the lowest potential level of market demand; inclusion of these other programs would serve to increase market demand above our lowest-level estimates.

**Table 1:** Total annual sites sampled (statewide and regional programs) per water body type.

	Average Annual No. of Sites								
State	Stream	Lake	Wetland	Source					
AK	34	10	0	http://dec.alaska.gov/water/wqsar/monitoring					
AL	700	0	0	https://fp.auburn.edu/icaae/biosites.aspx					
AR	17	0	0	http://www.adeq.state.ar.us/water					
AZ	100	16	0	http://www.azdeq.gov/environ/water					
CA	1411	0	87	http://www.waterboards.ca.gov					
CO	54	45	0	http://www.cdphe.state.co.us/op/wqcc					
CT	305	0	0	http://www.ct.gov/dep/lib/dep/water					
DE	360	27	0	http://www.dnrec.delaware.gov					
FL	386	4400	16	http://www.dep.state.fl.us/					
GA	120	0	0	http://www.georgiawildlife.com/node/734					
HI	0	0	0	http://www.pifsc.noaa.gov					
IA	88	393	0	http://www.igsb.uiowa.edu/wqm/Biological					
ID	250	0	0	http://www.deq.idaho.gov/media					
IL	100	0	0	http://www.epa.state.il.us/water/water-quality					
IN	311	0	0	http://www.in.gov/idem/files					
KS	123	38	2	http://www.kdheks.gov/befs					
KY	441	0	0	http://water.ky.gov					
LA	9	0	0	http://deq.louisiana.giv/portal					
MA	28	0	50	http://www.mass.gov/dfwele					
MD	782	0	345	http://www.dnr.state.md.us					
ME	50	0	25	http://www.maine.gov/dep/water/monitoring/biomonitoring					
MI	339	0	0	http://www.michigan.gov/documents/deq					
MN	423	0	95	http://www.pca.state.mn.us					
MO	80	0	0	http://www.dnr.mo.gov					
MS	38	0	0	http://www.deq.state.ms.us					
MT	49	0	0	http://www.deq.mt.gov/wqinfo/monitoring					
NC	160	0	0	http://portal.ncdenr.org/web/wq/benthosdata					
ND	30	10	0	http://www.ndhealth.gov/WQ/SW					
NE	39	4	0	http://www.deq.state.ne.us					
NH	50	35	37	http://des.nh.gov					
NJ	441	4	50	http://www.state.nj.us/dep/wms					
NM	34	18	0	http://www.nmenv.state.nm.us/					
NV	198	0	0	http://ndep.nv.gov/bwqp					
NY	497	0	0	http://www.dec.ny.gov					
OH	835	32	0	http://www.epa.state.oh.us/dsw/bioasses/ohstrat.aspx					
OK	547	104	0	http://www.owrb.ok.gov/quality/monitoring/bump.php					
OR	24	6	0	http://www.deq.state.or.us					
PA	127	0	0	http://files.dep.state.pa.us/Water					
RI	0	0	0	http://www.gso.uri.edu					
SC	80	0	30	http://www.dnr.sc.gov/marine/scecap/methods.html					
SD	175	148	0	http://denr.sd.gov					
TN	30	35	0	http://tn.gov/environment					
TX	2	0	0	http://www.cpcb.ku.edu/research/html/wadeablestreams.htm					
UT	85	5	0	http://www.waterquality.utah.gov/Monitoring					
VA	55	29	0	http://www.deq.state.va.us					
VT	164	10	0	http://www.vtwaterquality.org					
WA	143	55	40	http://www.dnr.wa.gov					
WI	487	291	0	http://dnr.wi.gov					
WV	150	0	0	http://www.dep.wv.gov/WWE/watershed					
WY	18	56	0	http://deq.state.wy.us/wqd/watershed					
All States	10969	5771	777						
Regional Programs	1846	199	14	see Table 2					
**Total**	**12815**	**5970**	**791**						

**Table 2:** Annual bioassessment effort by regional program.

Regional Programs	States Covered	Environment	Indicators	Annual Sites
Chesapeake Bay Program	DC, DE, MD, NY, PA, VA, WV	Stream	Benthic Macroinvertebrates	986
Delaware Estuary Program (DELEP)	DE, NJ, PA	Wetland	Benthic Macroinvertebrates	14
U.S. Army Corps of Engineers Louisville District	IN, KS	Stream	Benthic Macroinvertebrates	75
Ohio River Valley Water Sanitation Commission (ORSANCO)	IN, OH, PA, KY, WV	Stream	Fish	60
Susquehanna River Basin Commission Monitoring and Assessment Program	MD, NY, PA	Stream	Fish, Benthic Macroinvertebrates	153
Clark Fork-Pend Oreille Watershed Monitoring Program	MT, ID, WA	Stream	Benthic Macroinvertebrates, Algae	160
Delaware River Biomonitoring Program	NJ, NY, PA, DE	Stream	Benthic Macroinvertebrates, Algae	25
USGS National Water-Quality Assessment (NAWQA) Program	Eastern U.S.	Stream	Benthic Macroinvertebrates	109
USEPA Wadeable Streams Assessment (WSA)	National	Stream	Benthic Macroinvertebrates	278
Great Lakes Biological Open Water Surveillance Program	Great Lakes	Lakes	Benthic Macroinvertebrates, Algae	199

Sampling information for each state was first compiled by waterbody type, which included lakes, streams, and non-riverine wetlands. Next, the data was sorted by the three indicators of interest: algae, benthic macroinvertebrates, and fish, along with the taxonomic resolution stipulated by each program. Other indicators, such as amphibians, plankton, and plants that were included in some program assessments were noted, but not included in our analysis for purposes of this survey.

Level of effort was summarized based on the frequency and intensity of sampling. Frequency varied from weekly to once every decade. To facilitate comparison between states and programs, we standardized the data by calculating the average annual sampling intensity (i.e. how many sites and organisms were collected per year for each taxa). When organizations reported a range in the number of sample sites, we averaged the upper and lower estimates provided. Where sampling intensity was inconsistent over each year, approximations were made from multiple years' worth of data.

The number of organisms analyzed at each sampling site was estimated in one of three ways. When stipulated in agency reports, we used the number reported. If the precise number was not reported, but specific protocols were listed, we used those to obtain estimates of intensity. Where neither specific organism counts nor protocols were provided, we assumed that agencies relied on the United States Environmental Protection Agency's (USEPA) Rapid Bioassessment Protocols [15]. For benthic macroinvertebrates, the USEPA protocols recommend 200 benthic invertebrate specimens per sample (although actual sample counts could vary between 100 and 600 specimens/sample). For fish, we assumed 500 specimens per sample site based on electrofishing recommendations provided by the USEPA National River Streams and Assessment Field Operations Manual [16].

If information was not provided for the intensity of algal assessments, we relied on the State of California's Surface Water Ambient Monitoring Program (SWAMP) protocols, which are adapted from guidelines provided by the U.S. Geological Survey and a review of all other preceding state programs. These protocols stipulate a count of 600 diatom valves plus 300 soft-algal “entities” and 100 soft-algal epiphytes. We assumed 600 diatom valves only, unless otherwise specified.

We report on overall national level of effort as well as effort on a state-by-state basis. Information from the two major national bioassessment programs, administered by USEPA and USGS and nine other regional programs, were considered as part of the overall national level of effort. However, they were not included in the state-by-state summaries because specific sampling locations within the regional programs were not distinctly defined by state.

## Results

### Relative cost of traditional vs. molecular based taxonomy

We estimate that taxonomic analysis using current DNA barcoding approaches based on Sanger sequencing will cost between 1.7 and 3.4 times more than traditional morphology based approaches (Table 3). In contrast, use of next-generation methods results in cost parity with traditional approaches with costs being comparable to slightly lower depending on the indicator and the number of specimens analyzed (Table 3). The increased cost associated with Sanger sequencing is due mainly to the cost of DNA extraction, amplification, and sequencing compared to the cost sorting and identifying organisms based on morphological characteristics. Estimated costs for traditional morphology-based bioassessment of freshwater samples range from $600 to $850 per site excluding field sampling costs (Table 3). These costs reflect common protocols that are for 200 individuals per site for benthic invertebrates, 500 individuals per site for fish, and 600 diatom valves for algae, and involve taxonomy to genus or species level for most organisms. Between 50–60% of these costs are for the actual taxonomic identification, with the balance being for the initial picking and sorting of field samples in preparation for the actual analysis. Cost estimates from the 3–6 labs surveyed typically ranged between ± 30% of the median values presented in Table 3.

**Table 3:** Sampling and analysis cost of traditional vs. molecular based bioassessment.

		Traditional	Sanger	Next Gen (Ion Torrent)
***Field Sampling***				
	Fish	$1,500	$1,500	$1,500
	Invertebrates	$2,000	$2,000	$2,000
	Algae	$2,000	$2,000	$2,000
***Sorting (and clipping)***				
	Fish	$350	$400	$0
	Invertebrates	$450	$500	$0
	Algae	$300	$1,000	$0
***Taxonomic ID***				
	Fish	$500	$2,500	$500–$1000
	Invertebrates	$400	$1,000	$200–$400
	Algae (diatoms)	$300	$3,000	$600–$1,200
***Total cost (excluding field sampling)***				
	Fish	$850	$2,900	$500–$1000
	Invertebrates	$850	$1,500	$200–$400
	Algae	$600	$4,000	$600–$1,200
a.	molecular costs include extraction, amplification, and sequencing			
b.	algae molecular sorting includes cost of culturing pure strains			
c.	fish = 500 count			
d.	invertebrates = 200 count			
e.	algae = 600 diatom valves			

Median costs are presented. Range of reported costs was typically ±30%. NA  =  not applicable.

The cost for DNA barcoding using Sanger sequencing includes sorting and, when required, clipping tissue samples, and filling 96 well plates with whole vouchers or tissue from specimens grouped by compatibility of PCR primer treatments, and the actual molecular analysis. Sorting and clipping costs are comparable to the sorting costs for traditional taxonomic efforts, approximately $400–$600 per sample. The exception is for diatoms which require culturing pure strains prior to being able to extract DNA. This increases the overall cost to more than 6 times that of the traditional morphology-based approach. Average cost for molecular analysis using Sanger sequencing is approximately $5 per individual, with about half the cost associated with DNA extraction and amplification and the other half associated with sequencing. This cost assumes that DNA sequences can be obtained for all specimens in 1–3 attempts. If there are difficulties in obtaining acceptable sequences that meet quality standards, additional failure-tracking may be necessary, such as trying different primers or manual examination of sequences to look for errors. If necessary, these additional steps can result in a doubling of the cost to approximately $10 per individual sample. For a typical 200-count macroinvertebrate sample the best case, total cost for producing molecular-derived operational taxonomic units is approximately $1,500. At current rates this is approximately 1.7 times the cost of traditional morphology-based methods. The main variable that may affect these costs is the number of organisms analyzed per sample.

Barcoding programs are beginning to move toward next-generation sequencing (NGS), but this approach is still considered somewhat novel for this application. However, NGS costs are substantially less than those associated with Sanger sequencing because it is not necessary to sort specimens, clip tissues, and place extracts into individual wells on a plate. The cost for NGS based barcoding can be as low as fifty cents per individual specimen or as much as two dollars, and largely depends on the scale of the experiment and the platform being used [17]. Consequently, costs can be comparable (or slightly lower) than traditional taxonomic methods.

The increased cost associated with DNA barcoding is offset by substantial reduction in time necessary to receive results. Morphology-based methods typically require approximately six months to process samples and produce data that can be converted to biological indices. Approximately half this time is for sorting and the other half for taxonomic identification. In contrast, The Sanger sequencing approach can produce answers within three-five weeks (depends on analytical lab capacity), with approximately half the time necessary for sorting and tissue clipping and the rest for DNA extraction, amplification, and sequencing. The NGS approach using most recent platforms can produce answers in a matter of days. The shorter processing for next-generation methods results from higher throughput and the fact that sorting individual samples is not required.

### Assessment of market demand based on national level of effort

**Figure 1:**
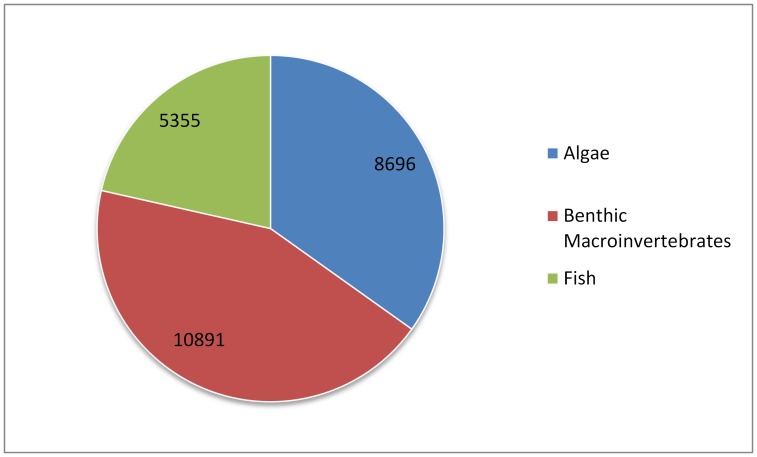
Annual sampling locations per indicator.

Substantial market demand is associated with bioassessment efforts in the U.S. Nationally, more than 19,500 sites are sampled for bioassessment purposes annually (Table 1). Streams are the most commonly sampled resource with more than 12,800 stream reaches sampled annually. Wetlands are the least sampled resource, with less than 800 sites sampled annually. Benthic macroinvertebrates are the most commonly used indicators, comprising almost half of the total sampling effort (Figure 1). Algae account for approximately 35% of total sampling effort and fish make up the remaining 21%.

**Figure 2:**
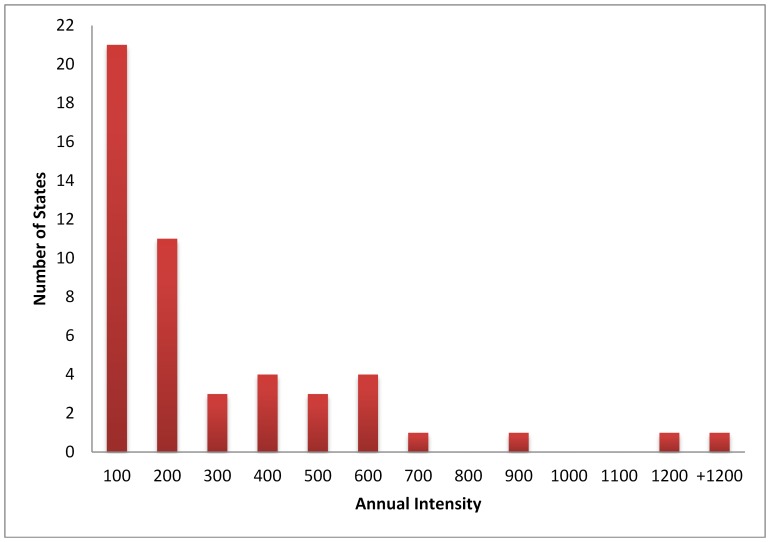
Distribution of annual intensity of sampling per state for all waterbody types combined.

Every state in the United States conducts some level of routine bioassessment monitoring; however, the level of effort varies widely (Figure 2). California and Florida have the largest programs, sampling 1,498 and 4,802 sites respectively, while 4 states sample less than 10 freshwater sites per year. Approximately 32 states sample 200 sites per year or less with only 2 states sampling more than 1,000 sites per year. Sampling intensity is typically highest along the east and west coasts and in the Midwest. State patterns mirror the nation in that the most common assessments are in streams using benthic macroinvertebrates (Table 4). However, algal assessments in streams are also fairly common, being used by 22 states. Lakes are the second most commonly sampled waterbody type with algae being the most common indicator sampled in lakes.

**Table 4:** Count of states that sample each water body type and indicator (does not include regional programs).

Habitat	Algae	Benthic Macroinvertebrates	Fish
Lake	19	9	7
Stream	22	45	30
Wetland	5	9	6

Regional programs account for approximately 11% of the total national sampling effort (Table 2). The Chesapeake Bay Program is the largest regional program, accounting for almost 1,000 sites sampled annually. As with the state programs, benthic macroinvertebrates are the most commonly used indicator. It is likely that there are many other local and regional programs across the country that we were not able to inventory through this effort. Therefore, the true effort and the local/regional level may be several times as large as what we estimated.

**Figure 3:**
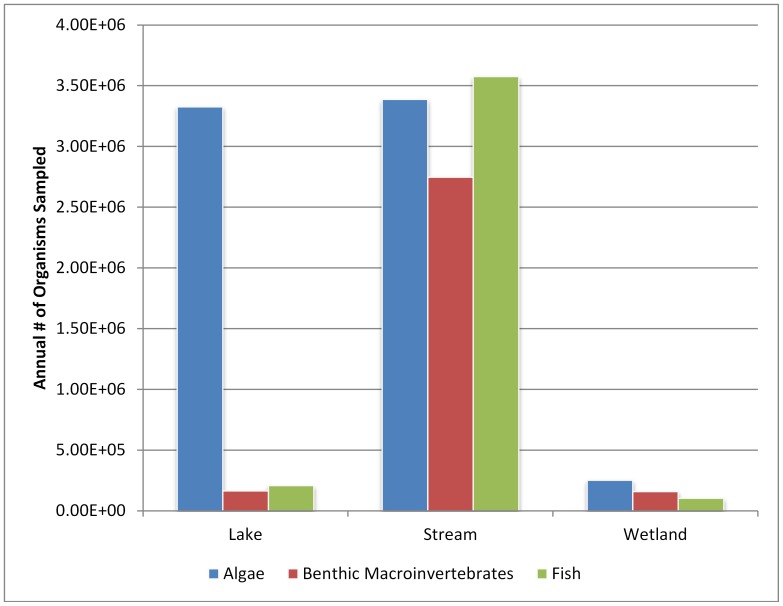
Annual number of organisms sampled (statewide and regional programs) per water body type and indicator.

Between 1.3–1.7×10^7^ organisms are collected annually across all programs for bioassessment purposes. Of these, approximately 7.0×10^6^ are algal valves and entities, 3.9×10^6^ are fish, and 3.1×10^6^ are benthic invertebrates. The high number of algal specimens collected may be an artifact of the assumption of 600 specimens per sample when the actual sample count is not specified. For benthic macroinvertebrates we assumed 200 specimens per sample were identified when sample counts were not specified. However, in reality this number may have ranged from 100 to 600 based on standard protocols. Therefore, the actual number of benthic invertebrates sampled could be between 2.2×10^6^ and 6.5×10^6^. In contrast, more detail is available on fish meaning those estimates are likely more reliable. Of the approximately 1.4×10^7^ organisms collected annually, 9.7×10^6^ are collected from streams, with other water body types contributing substantially less (Figure 3).

Based on the cost estimates in Table 3 and the reported sampling intensity, we estimate that between $104 and $193 million is spent annually on routine bioassessment in the United States (Table 5). Approximately 65% of these costs are spent by the six states with the largest programs (FL, CA, NY, OH, MD, and AL). Costs are spread across all aquatic habitats; however, stream monitoring is the largest contributor, accounting for approximately 58% of the expenditures. In terms of indicators, we estimate that costs are relatively evenly spread among benthic invertebrates, fish and algae. The cost to conduct traditional morphology-based taxonomy accounts for 30% of the bioassessment costs in freshwater. This translates to an annual expenditure on taxonomy of $31–$57 million, with approximately 64% of the expenditure occurring in streams. Benthic macroinvertebrates comprise approximately 36% of the annual expenditures on taxonomy.

**Table 5:** Annual bioassessment costs in millions of dollars by indicator and waterbody type.

	Lakes	Streams	Wetlands	Total
Fish	9.8–18.2	20.3–37.6	1.3–2.4	***31.4–58.2***
Benthic Invertebrates	11.9–22.2	24.6–45.6	1.6–2.9	***38.1–70.7***
Algae	10.9–20.2	22.4–41.6	1.4–2.7	***34.7–64.5***
*Total*	***32.6–60.6***	***67.3–124.8***	***4.3–8***	**104.2–193.4**

Costs include field sampling cost. NA  =  not applicable.

## Discussion

Based on current cost estimates, the promise of molecular methods as a less expensive alternative to traditional morphology-based taxonomy has not yet been realized. Current barcoding costs using the standard Sanger sequencing approach are between 1.7 and 3.4 times the cost of traditional taxonomic identification and could be as much as ten times the cost if significant failure-tracking and re-sequencing is required. Our cost estimates are similar to the $5 per sample costs reported by Cameron et al. [18] and the $3–$7.50 per sample for plants reported by [19], both of which included the cost of PCR, purification, and sequencing. The additional overall costs associated with Sanger sequencing (relative to NGS) results from the need to still pick, sort and clip individual specimens prior to barcoding. These costs do not include the up-front cost of building a local reference library of fully vouchered specimens with barcodes, which may or may not be necessary depending on the type of bioassessment metrics used. Some bioassessment scoring tools include functional or trait-based metrics that require knowing the identity of individual species so that tolerance values or trait attributions can be assigned [20]–[21]. If species identities are necessary, a local reference library will typically be required to augment the species identity information available in the Barcode of Life Database. If ten replicate voucher specimens were obtained for each taxa in the library, the additional cost of library development would be approximately $50,000 per 1,000 taxa, excluding field sampling costs. Although not trivial, this cost is relatively low compared to typical investments in monitoring programs. Bioassessment scoring tools that rely mainly on diversity or richness-based metrics do not require information on individual species identities, instead they only require knowing the number of species present. In this case, the Operational Taxonomic Units derived from raw sequences using Sanger sequencing is sufficient and a local reference library would not be necessary. However, identifying the number of OTUs from NGS may be challenging due to issues such lumping/splitting of OTUs and amplification bias; therefore, more research is needed before NGS results could be directly applied to bioassessment. For NGS methods, there may be additional costs associated with establishing the bioinformatics pipeline necessary to analyzing the large quantities of data produced by these methods [17].

The cost-effectiveness, or at least cost parity of DNA barcoding has been promoted as one of the benefits of incorporating molecular methods into routine biomonitoring programs [22]. These estimates are based on the assumption that barcoding would cost between $2 and $5 per sample, after the picking and sorting has been completed. Although these estimates appear reasonable based on our analysis, our direct comparison indicates that traditional morphology-based taxonomy may still be less expensive for freshwater bioassessment. The relative cost of barcoding depends partly on the type of sequencing that is used. Sanger sequencing is more costly, but provides higher accuracy and allows for direct linkage between individual sequences and the specimen they were taken from. This is important during development of new assessment tools, for detailed traits-based analysis, or when using barcoding as a quality control check for taxonomic identification. Next-generation methods are currently less accurate (due to shorter sequence lengths and amplification biases) and do not allow linkage back to the specimen of origin. However, the cost for an NGS run can be less than $1 per specimen, which is commensurate with the cost of morphology-based identifications.

An important consideration when comparing the cost of morphology and molecular methods is that molecular methods require additional data processing in order to convert sequence data to “Operational Taxonomic Units” or “putative species” that can be used to calculate bioassessment metrics. This process may be automated for sequences stored and managed by the Barcode of Life Database, but NGS derived sequence data will require extensive processing before it can be used for bioassessment. In contrast, the results provided by traditional taxonomic analysis can be directly used to calculate metrics. These data processing costs are not included in our analysis. In general, data processing costs will be higher for NGS than for Sanger sequencing given the high throughput and volume of data produced by next-generation methods.

Although current barcoding approaches are more expensive, they do provide additional benefits of being able to obtain answers in substantially less time. For example, in the time it takes to complete traditional taxonomic analysis, 3–4 times as much barcoding could be done. The reduced time to obtain answers could also allow for easier adaptive management of monitoring and could facilitate use of biological indicators in situations where a more immediate answer is necessary, such as environmental damage assessment following spills. Furthermore, as shown by others, the increased taxonomic resolution provided by DNA barcoding can improve the sensitivity and performance of commonly used bioassessment metrics [19], [23]–[26].

As technology continues to advance, costs associated with molecular methods will continue to drop. Emerging approaches such as bulk sampling, whereby picking and sorting of individuals is not necessary may reduce costs even further. Bulk sampling involves processing large volumes of composite samples in a mixed matrix, extracting the DNA in bulk and producing a list of component species [27]. This is in contrast to the sample-by-sample analysis required by standard Sanger sequencing. Recent studies have shown that it is possible to detect a wide range of taxonomic groups from environmental DNA isolated directly from water samples within which and downstream of where target organisms reside [28]. Analytical cost and time may be further reduced by improvements in next-generation high throughput sequencing and metagenomic methods that don't require PCR amplification [29]. These, and other yet unforeseen advances will likely lead to cost-effectiveness complementing other benefits of DNA barcoding.

We believe that there is sufficient market demand to justify continued investment in the application of molecular methods to routine bioassessment, despite the fact that cost parity is yet to be realized. Our results suggest that more than 19,500 freshwater sites are sampled annually, accounting for over 13 million specimens analyzed, the majority of which are benthic macroinvertebrates. This is consistent with the findings of Ruaro and Guiani [2] who reported that the vast majority of monitoring programs rely on macroinvertebrates despite the fact that more than half the papers they reviewed focused on IBI development for fish. Our estimates of the magnitude of stream bioassessment for macroinvertebrates are approximately 10% lower than those reported by Carter and Resh [11]. This may be due to the fact that they used direct surveys vs. our more passive Internet search and/or that their survey included additional types of bioassessment effort (see below).

The level of effort and cost estimates provided here certainly underestimate actual annual expenditures on bioassessment and thus represent a basement (or lowest level) estimate of actual market demand. Our analysis was limited to routine ambient assessment programs run by state, regional or federal agencies where information is readily available. However, bioassessment efforts are much more pervasive. County or local level monitoring programs were not included in our estimates. Compliance monitoring programs often include bioassessment, and several states have developed or are developing regulatory biocriteria based on bioassessment endpoints [30]. Bioassessment may also be used as part of routine monitoring for timber or agricultural operations, mine site assessment, or spill response damage assessment. Performance monitoring for stream or wetland restoration or site remediation may also include bioassessment indicators. Finally, citizen monitoring programs and tribal programs often include bioassessment, but this data is not readily available. Collectively, these additional programs could easily represent 10,000–25,000 additional annual monitoring sites, assuming they include 100–250 additional sites per state (based on rough estimates from California). This would translate to an additional 2–5 million specimens analyzed annually. Furthermore, our study did not include a survey of marine monitoring programs because they are much less consistently documented nationwide. These programs may contribute several thousand additional sites and several hundred thousand additional specimens. For example, the Southern California Bight and San Francisco Bay marine monitoring programs in California collectively sample over a 1,000 sites/year, and the Chesapeake Bay program along the east coast of the U.S. samples several hundred sites/year.

Although our study focused on the U.S., bioassessment efforts also occur in other parts of the world. A recent review of monitoring programs associated with European Union's (EU) Water Framework Directive indicated that bioassessment is a common tool for environmental evaluation in the EU, with 28 countries reporting on methods applied to rivers, coastal waters, lakes, and transitional waters. Of the methods reported on in the EU, more than half are based on macroscopic plants or benthic invertebrates; other indicators include phytoplankton, fish and phytobenthos [31]. One of the few surveys of bioassessment effort for Australia reported that the Australia-wide Assessment of River Health assessed 1,100 sites over a six year period ending in 2000 [32]. Consequently, it is likely that global levels of effort devoted to bioassessment are at least as great as we observed in the U.S. and therefore cost considerations will likely be commensurate across the globe.
